# Saikosaponin A alleviates *Staphylococcus aureus*‐induced mastitis in mice by inhibiting ferroptosis via SIRT1/Nrf2 pathway

**DOI:** 10.1111/jcmm.17914

**Published:** 2023-08-29

**Authors:** Lihua Zhao, Lei Jin, Bin Yang

**Affiliations:** ^1^ Department of Breast Surgery China‐Japan Union Hospital of Jilin University Changchun China; ^2^ Department of Anesthesiology China‐Japan Union Hospital of Jilin University Changchun China

**Keywords:** ferroptosis, mastitis, NF‐κB, *S. aureus*, Saikosaponin A

## Abstract

Mastitis is a common and serious bacterial infection of the mammary gland. Saikosaponin A (SSA) is a triterpenoid saponin isolated from *Bupleurum falcatum* that has the ability to treat various diseases. However, little is known about the role of SSA in achieving mastitis remission. Here, we found that SSA alleviated *Staphylococcus aureus* (*S. aureus*)‐induced mastitis by attenuating inflammation and maintaining blood‐milk barrier integrity. Furthermore, *S. aureus* activated nuclear factor kappa B (NF‐κB) pathway by upregulated p‐p65 and p‐IκB. *S. aureus* also induced ferroptosis in mammary gland in mice, mainly characterized by excessive iron accumulation, mitochondrial morphological changes and impaired antioxidant production. However, *S. aureus*‐induced NF‐κB activation and ferroptosis were prevented by SSA. Moreover, SAA could upregulate the expression of SIRT1, Nrf2, HO‐1 and GPX4. And the inhibitory effects of SAA on inflammation and ferroptosis were reversed by SIRT1 inhibitor EX‐527. In conclusion, SAA protected *S. aureus*‐induced mastitis through suppressing inflammation and ferroptosis by activating SIRT1/Nrf2 pathway.

## 1 INTRODUCTION

Mastitis is a common infectious disease in humans and animals, causing significant economic losses in the dairy industry and affecting the quality and safety of dairy products.^1^, ^2^ There are many causes of mastitis, and pathogenic microbial infection is one of the most common causes.^3^ *Staphylococcus aureus* (*S. aureus*) is one of the main pathogenic bacteria. Due to its characteristics of intracellular parasitize and immune escape, it is difficult to remove once invading the mammary gland.^4^ Therefore, *S. aureus*‐induced mastitis is very difficult to cure. Currently, antibiotics are still the main treatment for mastitis caused by *S. aureus* in clinical practice, but the overuse of antibiotics will lead to drug residues and bacterial resistance. Besides, antibiotics had no significant effect on the bacteria clearance of mammary gland and the repair of the blood‐milk barrier.^5^ Therefore, there is an urgent need to identify alternative therapeutic agents for the treatment of *S. aureus*‐induced mastitis.

Saikosaponin A (SSA) is a triterpenoid saponin extracted from the medicinal plant *Bupleurum falcatum*.^6^, ^7^ SSA has been reported to have various pharmacological activities, including anti‐inflammatory,^8^ antioxidant^9^ and anticancer effects.^10^ In primary mouse macrophages, SSA can inhibit LPS‐induced TNF‐α and IL‐1β production.^11^ SSA has also been demonstrated to alleviate CCL4‐induced acute hepatocellular injury by inhibiting oxidative stress and activation of inflammasome.^12^ In addition, SSA is considered to have a specific inhibitory effect on nuclear factor kappa B (NF‐κB) activation.^9^ For example, SSA can significantly relieve neuropathic pain by inhibiting the NF‐κB pathway and p38 MAPK activation in rats.^13^ It has also been shown that SSA alleviated hyperlipidemic pancreatitis by activating peroxisome proliferator‐activated receptor γ (PPARγ) expression and inhibiting NF‐κB inflammatory pathway.^14^ NF‐κB signal pathway is also involved in the occurrence and development of mastitis. Ran et al.^15^ found that dioscin improved LPS‐induced mastitis by inhibiting NF‐κB pathway and activating AMP‐activated protein kinase (AMPK)/nuclear factor erythroid‐2‐related factor 2 (Nrf2). Similarly, probiotic *Enterococcus mundtii* H81 inhibits the NF‐κB pathway to mitigate mastitis caused by *S. aureus* in mice.^16^ However, whether SSA can ameliorate *S. aureus*‐induced mastitis by inhibiting the NF‐κB pathway remains to be further investigated.

Ferroptosis is a newly discovered form of cell death that is different from apoptosis, necrosis and autophagy.^17^ It is characterized by mitochondrial atrophy, lipid peroxidation, iron accumulation, heightened levels of prostaglandin endoperoxidase 2 (PTGS2) and reduced glutathione peroxidase 4 (GPX4).^17^ Ferroptosis has been implicated in various diseases, including cancer,^18^ neurodegeneration^19^ and inflammatory disease.^20^ Zhang et al. demonstrated that ferroptosis was involved in clinical mastitis in dairy cows and heme oxygenase 1 (HMOX1) promoted ferroptosis in mammary epithelial cells via FTH1.^21^ IL‐6 promoted ferroptosis and inflammation through the Nrf2 signalling pathway in goat mammary epithelial cells.^22^ Besides, NF‐κBp65 phosphorylation inhibited ferroptosis to alleviate ulcerative colitis.^23^ And ferrostatin‐1 (Fer‐1), a ferroptosis inhibitor, significantly reduced the level of toll‐like receptor 4 (TLR4), phospho‐nuclear factor kappa B (NF‐κB) and phospho‐inhibitor of kappa Bα (IκBα) in rats.^24^ Notably, the effect of SSA on ferroptosis and whether SSA can alleviate *S. aureus*‐induced mastitis in mice by regulating ferroptosis remain unclear.

In this study, we found that SSA significantly ameliorated *S. aureus*‐induced mastitis by inhibiting ferroptosis and inflammation. The results suggest that SSA may be a promising therapeutic agent for the prevention and treatment of *S. aureus*‐induced mastitis, as well as other inflammatory diseases associated with ferroptosis.

## 2 MATERIALS AND METHODS

### 2.1 Animals

All BALB/c mice (21–25 g, 6–8 weeks old) were provided by Liaoning Changsheng. They were given plenty of water and food and placed in a specific pathogen‐free facility with a 12‐h light and dark cycle. All mice experiments were conducted abide by and approved by the IACUC of Jilin University. To establish a *S. aureus*‐induced mastitis model, 1 × 10^8^ CFU/mL *S. aureus* 100 μL was injected into the udder canals of L4 (left) and R4 (right), and tissue samples were collected 24 h later. SSA (5, 10 and 20 mg/kg) was injected intraperitoneally 1 h before *S. aureus* treatment. The doses of SSA used in this study were based on previous study.^25^

### 2.2 Reagents

Saikosaponin A (purity > 98%) was obtained from Sigma. ELISA kits were purchased from BioLegend. The antibodies were obtained from Affinity Biosciences. GPX4 was gained from Bioss. EX‐527 was purchased from Selleck Chemicals.

### 2.3 Bacteria cultures

*Staphylococcus aureus* (ATCC35556) was obtained from ATCC. *S. aureus* was cultured in tryptic soy broth medium at 37°C 180 r/min for 8 h to reach the mid‐log phase.

### 2.4 Haematoxylin and eosin staining

The mammary samples were harvested and immobilized with 4% paraformaldehyde for more than 48 h. Then, these samples were embedded in paraffin to prepare 4 μm sections and stained with haematoxylin and eosin. Histopathological changes were observed by optical microscopy (Olympus), and the histological score was as previously described.^26^

### 2.5 ELISA

The content of proinflammatory cytokine in the mammary gland was detected by ELISA assay kit according to the manufacturer's instructions. The absorbance values were read at 450 nm and 570 nm by an automated enzyme standard instrument.

### 2.6 Western blots analysis

Proteins were extracted using protein extract, and protein concentrations were measured by bicinchoninic acid method. The proteins (30 μg) were separated using 12% SDS‐PAGE and then the proteins to a PVDF membrane following methanol treatment. The PVDF membranes were blocked in 5% skim milk and then incubated with specific primary and secondary antibodies. Finally, the proteins were visualized using an enhanced chemiluminescence solution and were detected with the ECL system.

### 2.7 Measurement of myeloperoxidase, glutathione, malondialdehyde and iron

Total glutathione (GSH) in tissue lysates was measured with GSH detection kit according to the manufacturer's instruction. The content of myeloperoxidase (MPO) and malondialdehyde (MDA) in tissue was measured with detection kits according to the manufacturer's instruction. Total Fe and Fe^2+^ release levels were determined using iron assay kit according to the manufacturer's instructions.

### 2.8 Statistical analysis

All data are showed as the means ± SEM and analysed using one‐way anova (Dunnett's *t*‐test). The data are considered statistically significant at *p* < 0.05 or *p* < 0.01.

## 3 RESULTS

### 3.1 Saikosaponin A alleviates *Staphylococcus aureus*‐induced mammary histological injury

**FIGURE 1:**
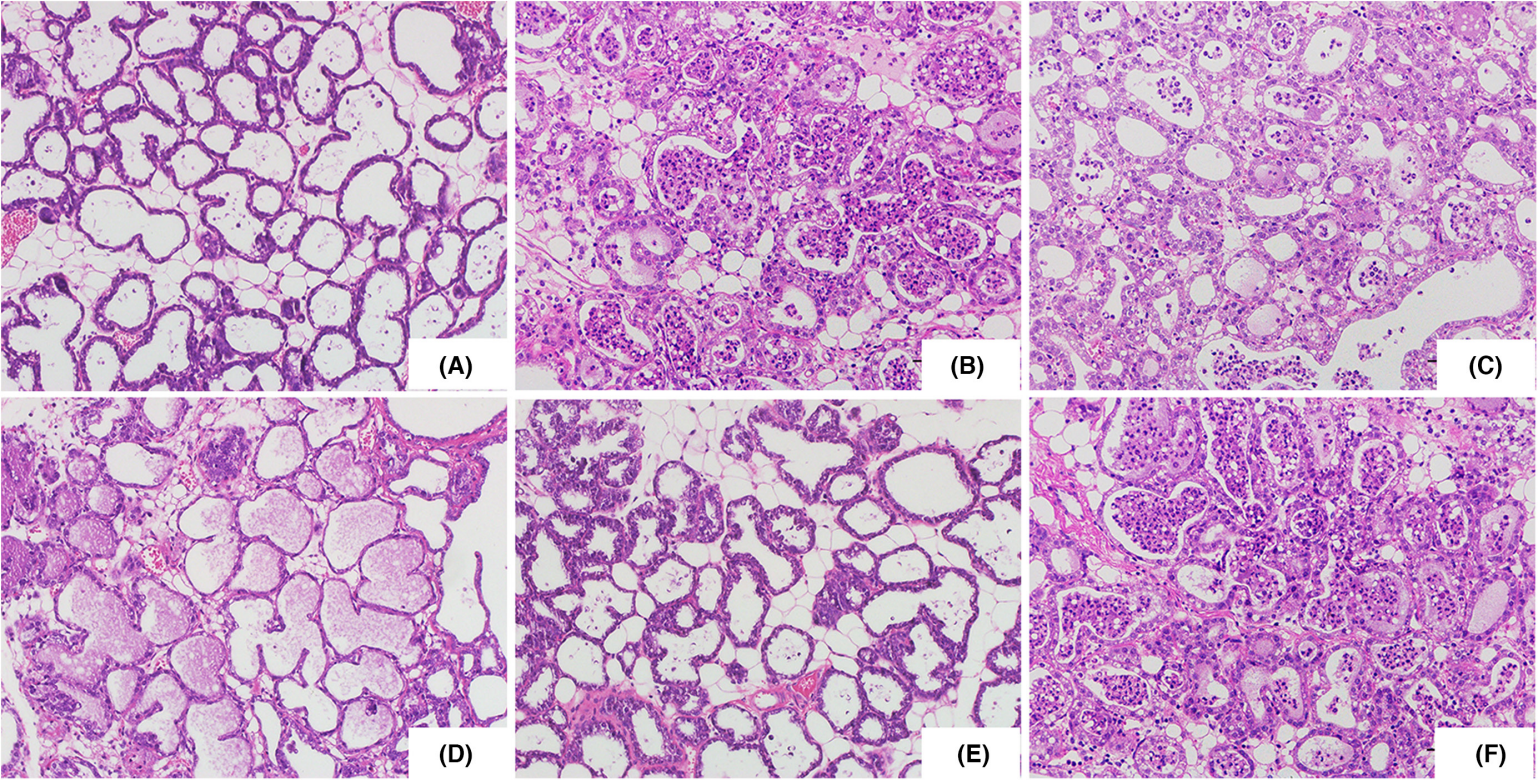
Effects of Saikosaponin A (SSA) on *Staphylococcus aureus* (*S. aureus*)‐induced mammary histopathological changes. Histopathologic sections of mammary tissues (haematoxylin and eosin, ×100). (A) control, (B) *S. aureus*, (C) *S. aureus* + SSA (5 mg/kg), (D) *S. aureus* + SSA (10 mg/kg), (E) *S. aureus* + SSA (20 mg/kg), (F) *S. aureus* + SSA (20 mg/kg) + EX527 (10 mg/kg).

To investigate the therapeutic effects of SSA on *S. aureus*‐induced mastitis in mice, we conducted a dose‐dependent study using various doses of SSA (5, 10 and 20 mg/kg). The results showed that SSA treatment significantly alleviated *S. aureus*‐induced mammary damage, which was mainly manifested as inflammatory cell infiltration and structure destruction (Figure 1). However, the protective effects of SAA on *S. aureus*‐induced mammary histological injury were prevented by SIRT1 inhibitor EX‐527 (Figure 1).

### 3.2 Saikosaponin A alleviates *Staphylococcus aureus*‐induced inflammatory response

**FIGURE 2:**
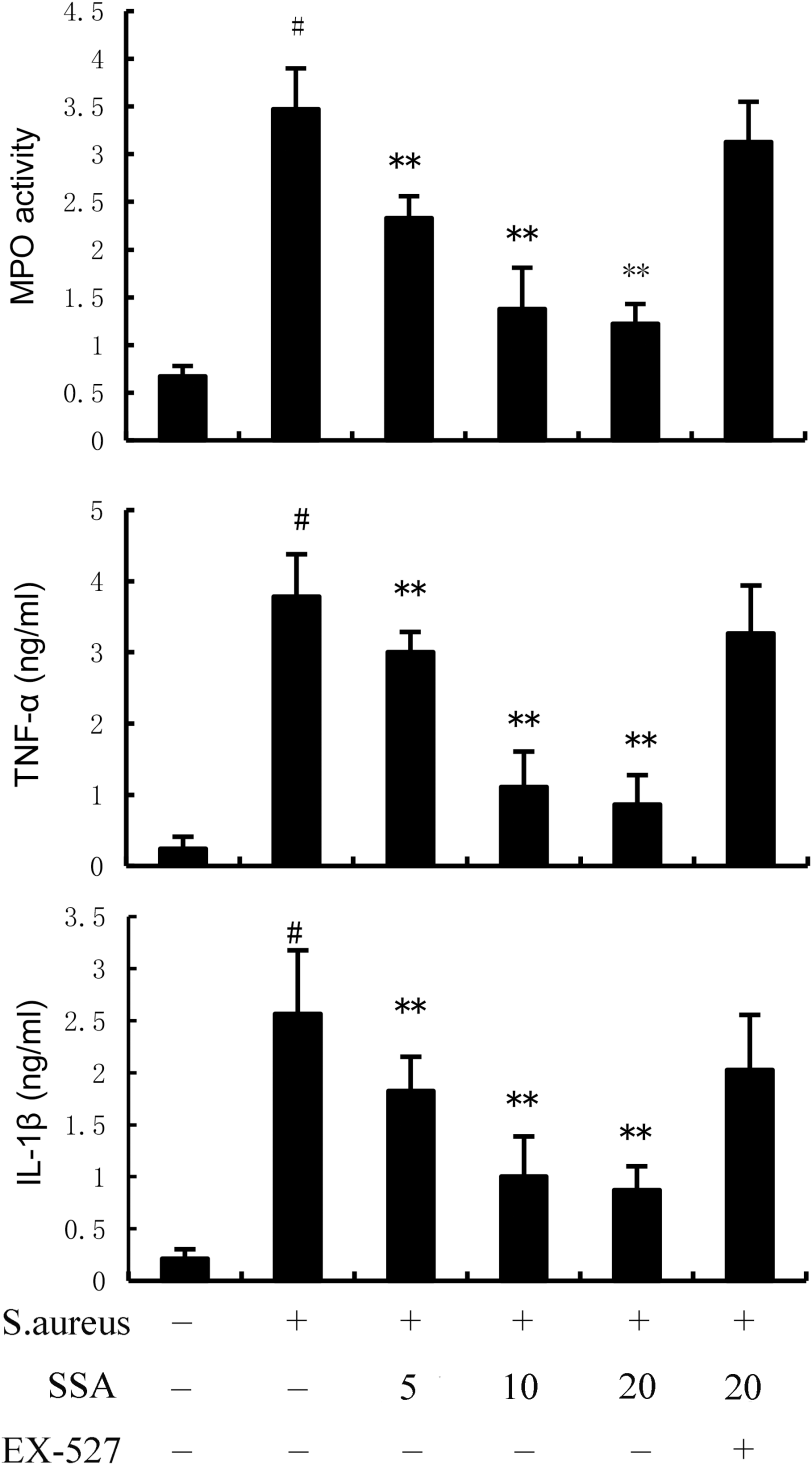
Effect of Saikosaponin A (SSA) on MPO activity and inflammatory cytokine production in mammary gland. The values presented are the mean ± SEM. ^#^ *p* < 0.01 is significantly different from control group; **p < 0.01 is significantly different from *Staphylococcus aureus* (*S. aureus*) group.

MPO activity and inflammatory cytokine production were tested to assess mammary inflammatory level. As demonstrated in Figure 2, MPO activity, TNF‐α and IL‐1β increased markedly in *S. aureus*‐treated mice. Likewise, SSA markedly reduced the elevated levels of inflammatory markers caused by *S. aureus*, including MPO, TNF‐α and IL‐1β (Figure 2). However, the inhibitory effects of SAA on *S. aureus*‐induced inflammation were prevented by SIRT1 inhibitor EX‐527 (Figure 2).

### 3.3 Saikosaponin A improves *Staphylococcus aureus*‐induced blood‐milk barrier injury in mice

**FIGURE 3:**
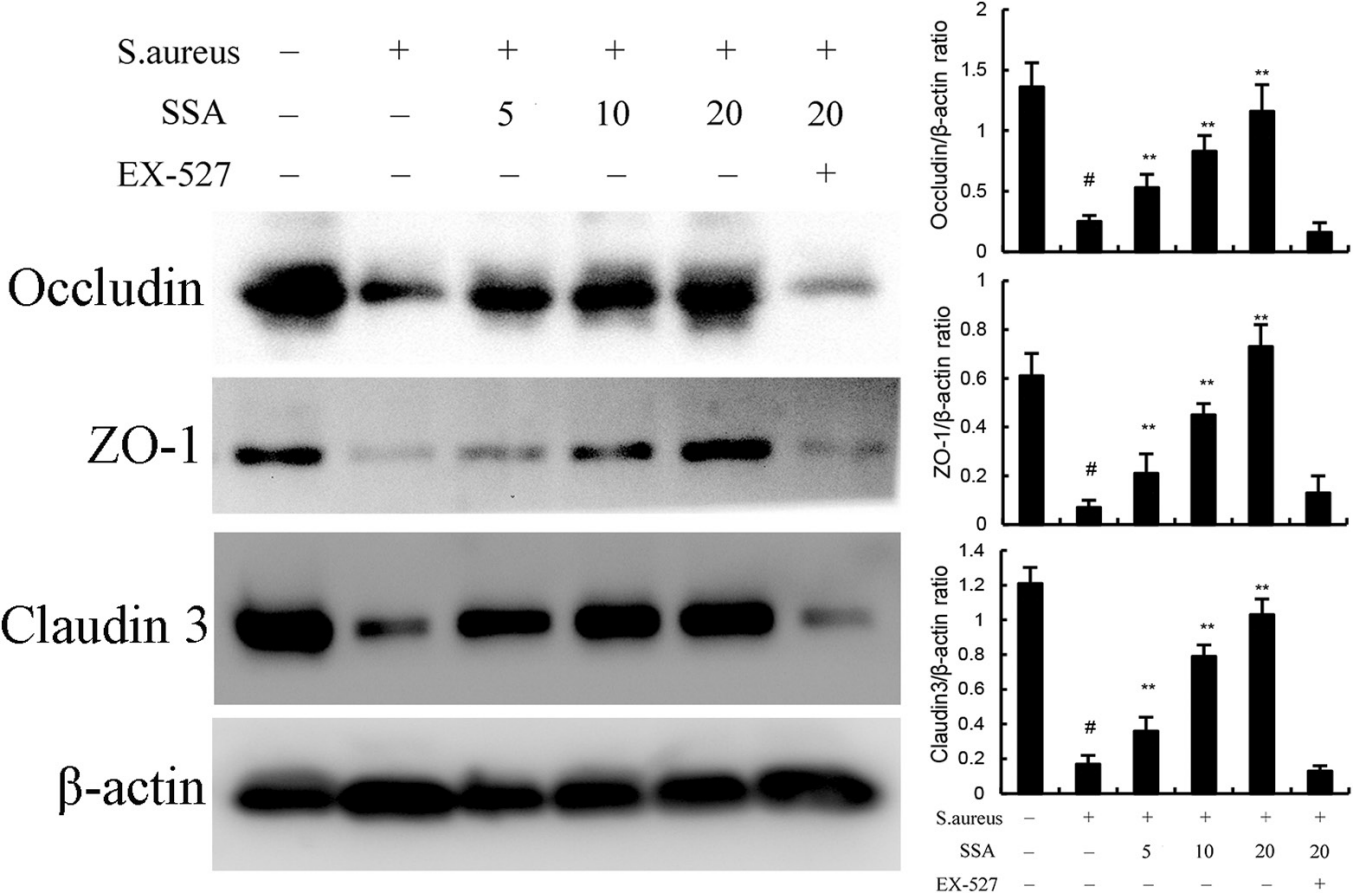
Effect of Saikosaponin A (SSA) on tight junction expression in mammary gland. The values presented are the mean ± SEM. ^#^ *p* < 0.01 is significantly different from control group; **p < 0.01 is significantly different from *Staphylococcus aureus* (*S. aureus*) group.

Studies have shown that overexpression of proinflammatory cytokines contributes to the destruction of the tight junction (TJ).^27^, ^28^, ^29^ Therefore, we investigated whether SSA can repair blood‐milk barrier damage induced by *S. aureus*. We found that the levels of TJ proteins including ZO‐1, occludin and claudin‐3 were reduced in *S. aureus‐*treated group (Figure 3). However, SSA significantly reversed these changes (Figure 3). Taken together, these results suggest that SSA ameliorates blood‐milk barrier damage induced by *S. aureus*. Furthermore, the inhibitory effects of SAA on *S. aureus*‐induced TJ injury were prevented by SIRT1 inhibitor EX‐527 (Figure 3).

### 3.4 Saikosaponin A improves *Staphylococcus aureus*‐induced ferroptosis in mice

**FIGURE 4:**
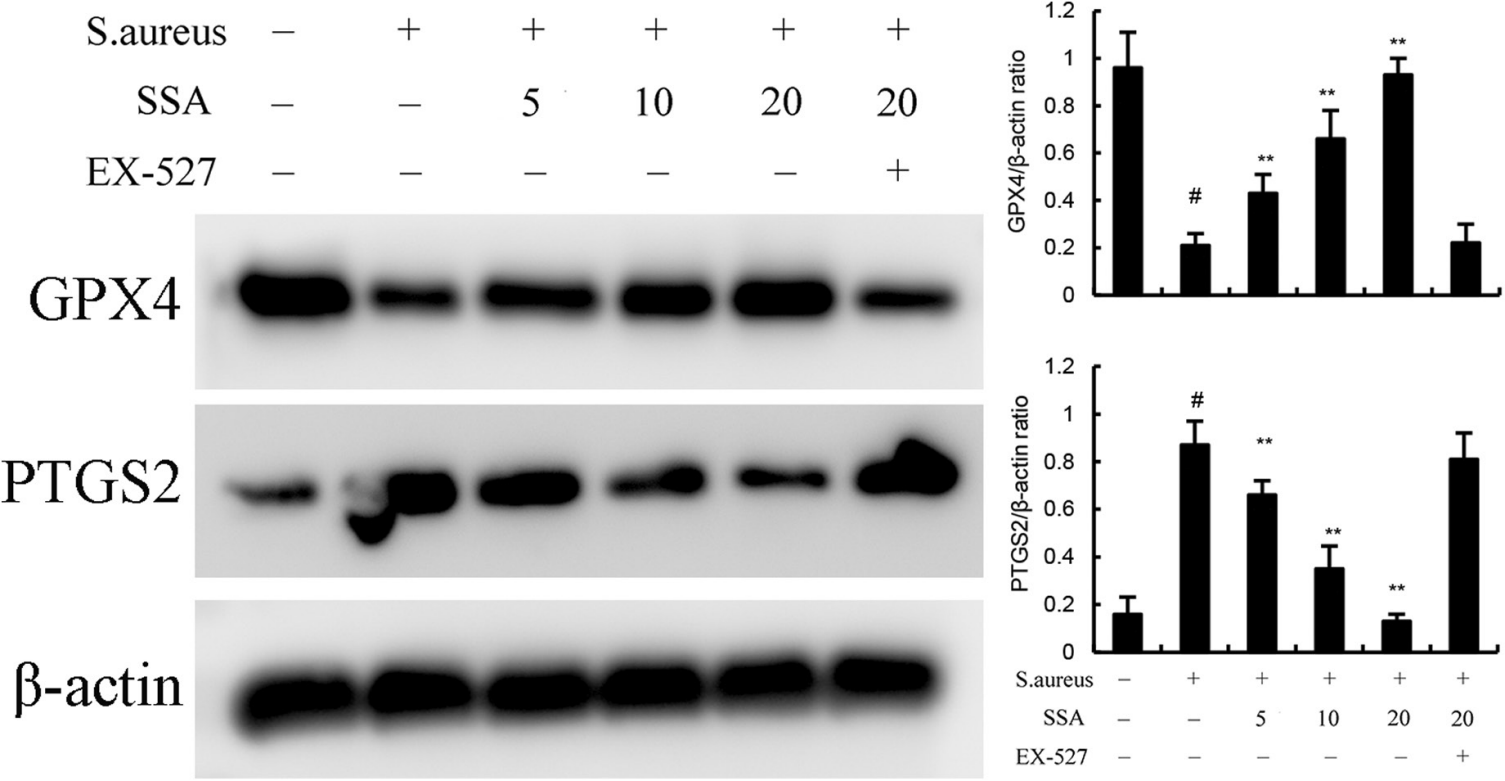
Effect of Saikosaponin A (SSA) on GPX4 and PTGS2 expression in mammary gland. The values presented are the mean ± SEM. ^#^ *p* < 0.01 is significantly different from control group; **p < 0.01 is significantly different from *Staphylococcus aureus* (*S. aureus*) group.

**FIGURE 5:**
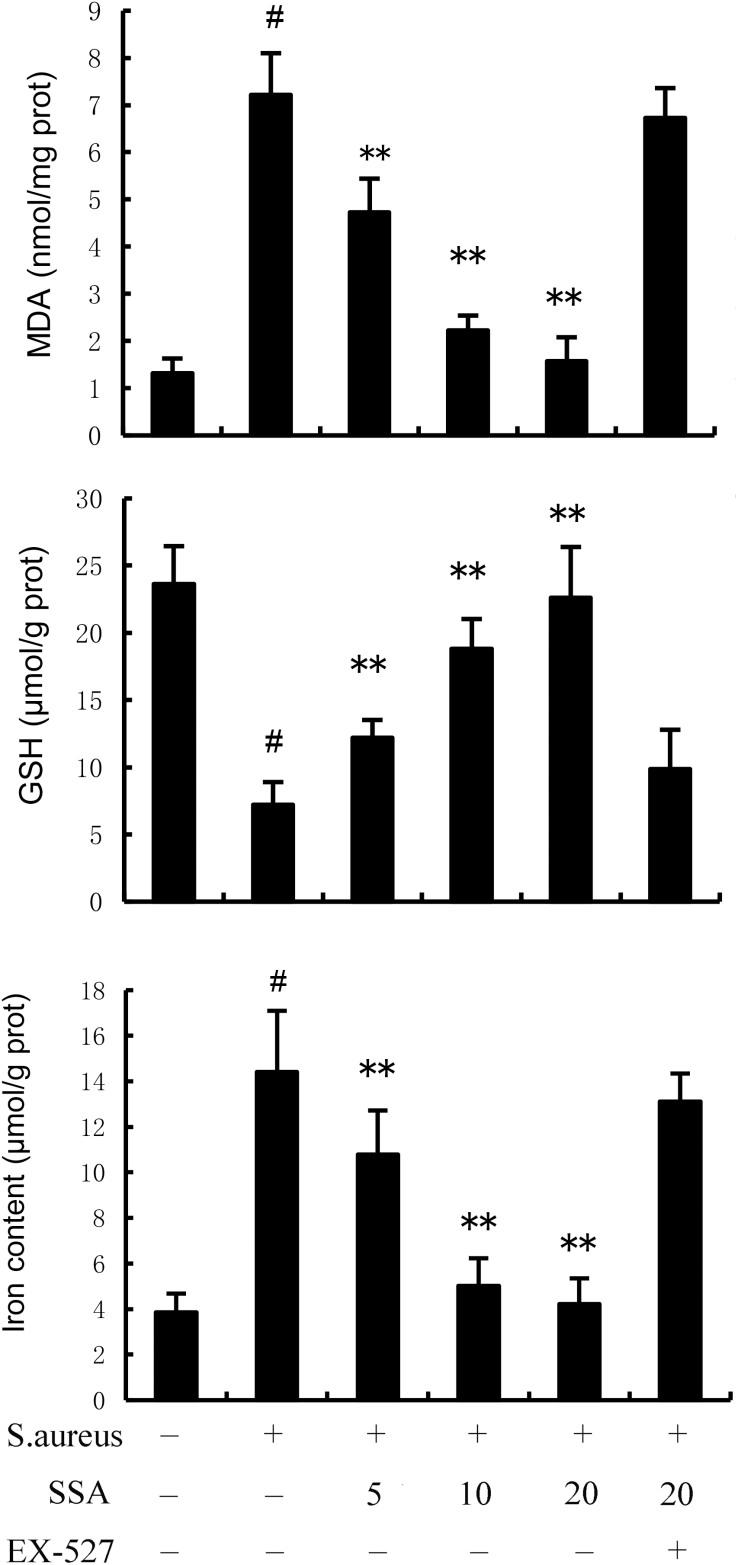
Effect of Saikosaponin A (SSA) on glutathione (GSH), iron and MDA production in mammary gland. The values presented are the mean ± SEM. ^#^ *p* < 0.01 is significantly different from control group; **p < 0.01 is significantly different from *Staphylococcus aureus* (*S. aureus*) group.

To confirm the effects of SSA on ferroptosis, we examined the protein expression levels of PTGS2 and GPX4. The results showed that *S. aureus* significantly increased the level of PTGS2 but decreased the level of GPX4 (Figure 4). Iron accumulation is one of the main characteristic of ferroptosis, so we examined the iron content in mammary gland. We observed that *S. aureus* treatment markedly increased the level of Fe^2+^ compared with untreated group (Figure 5). Meanwhile, *S. aureus*‐treated mice showed higher MDA expression and lower GSH expression (Figure 5) than untreated mice. These results suggested *S. aureus* could induce ferroptosis in mammary gland tissue. However, SAA markedly alleviated *S. aureus*‐induced ferroptosis (Figures 4, 5). Furthermore, the inhibitory effects of SAA on *S. aureus*‐induced ferroptosis were prevented by SIRT1 inhibitor EX‐527 (Figures 4, 5).

### 3.5 Saikosaponin A attenuates *Staphylococcus aureus*‐induced NF‐κB pathway in mice

**FIGURE 6:**
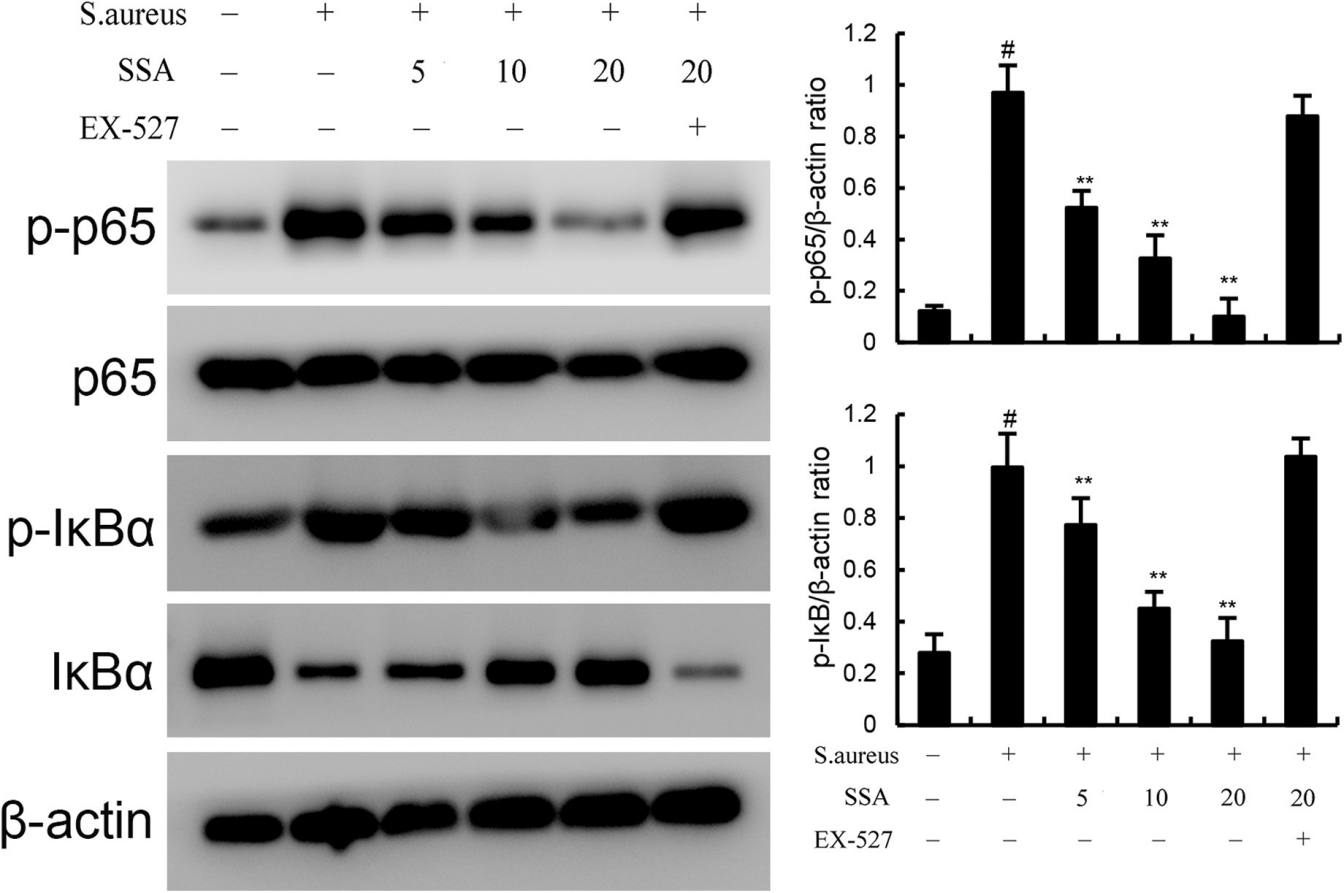
Effect of Saikosaponin A (SSA) on NF‐κB activation in mammary gland. The values presented are the mean ± SEM. ^#^ *p* < 0.01 is significantly different from control group; **p < 0.01 is significantly different from *Staphylococcus aureus* (*S. aureus*) group.

We next investigated the role of NF‐κB pathway in *S. aureus*‐induced mastitis in mice. Western blotting was used to detect the activation of NF‐κB pathway and found that *S. aureus* increased the protein levels of phosphorylated p65 and IκB (Figure 6). *S. aureus* activates the NF‐κB pathway in mice. Treatment of SSA suppressed *S. aureus*‐induced NF‐κB activation markedly (Figure 6). However, the inhibitory effects of SAA on *S. aureus*‐induced NF‐κB activation were prevented by SIRT1 inhibitor EX‐527 (Figure 6).

### 3.6 Saikosaponin A inhibits *Staphylococcus aureus*‐induced inflammation and ferroptosis by activating SIRT1/Nrf2 signal pathway

**FIGURE 7:**
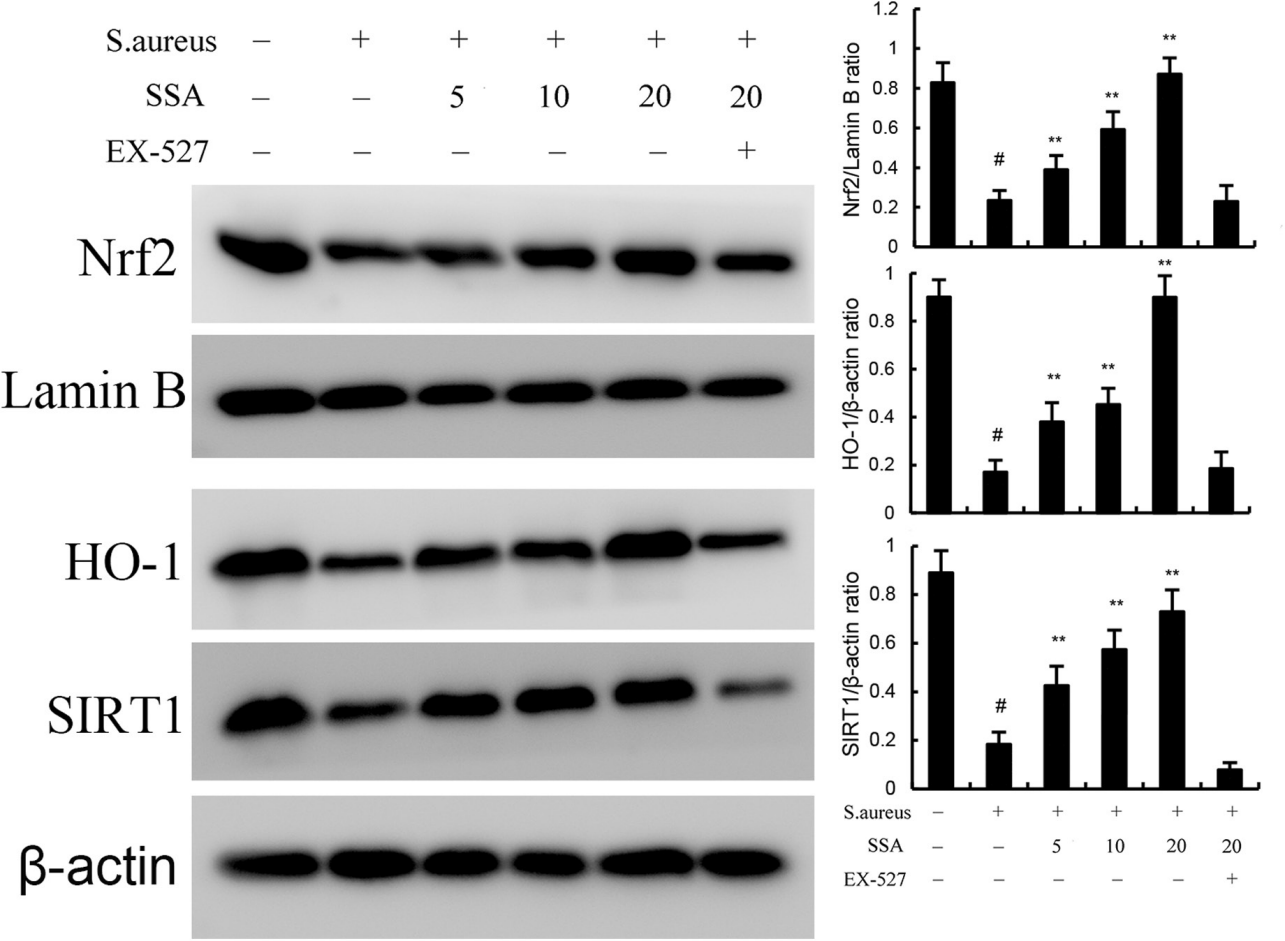
Effect of Saikosaponin A (SSA) on SIRT1, Nrf2 and HO‐1 expression in mammary gland. The values presented are the mean ± SEM. ^#^ *p* < 0.01 is significantly different from control group; **p < 0.01 is significantly different from *Staphylococcus aureus* (*S. aureus*) group.

Nrf2 was involved in inflammation and ferroptosis. In this study, expression of SIRT1, Nrf2 and HO‐1 was decreased by *S. aureus*. Treatment of SSA increased the expression of SIRT1, Nrf2 and HO‐1 in a dose‐dependent manner (Figure 7). However, the upregulation of SAA on SIRT1, Nrf2 and HO‐1 expression was prevented by SIRT1 inhibitor EX‐527 (Figure 7). Meanwhile, we found the inhibition of SSA on inflammation and ferroptosis were reversed by SIRT1 inhibitor EX‐527. These data suggested SAA inhibited *S. aureus*‐induced mastitis by activating SIRT1/Nrf2 signal pathway.

## 4 DISCUSSION

Mastitis is a common infectious disease in dairy cows, causing significant economic losses in the dairy industry and affecting the quality and safety of dairy products.^30^ *S. aureus* is one of the major causative agents of mastitis in dairy cows.^31^ It has been shown that the NF‐κB pathway is involved in the pathological process of *S. aureus*‐induced mastitis.^32^ Moreover, the NF‐κB pathway has been associated with ferroptosis.^33^ However, the role of SSA in ferroptosis and *S. aureus*‐associated mastitis and the underlying mechanisms are still unknown. In this study, we found that SSA could alleviate *S. aureus*‐induced mastitis in mice. SSA treatment improved blood‐milk barrier integrity, reduced mammary gland damage and inhibited ferroptosis in mammary gland. Moreover, SSA treatment also inhibited the activation of the NF‐κB pathway during *S. aureus*‐induced mastitis. These findings suggest that SSA could be a potential therapeutic agent for the treatment of *S. aureus*‐induced mastitis.

Inflammation is the host's protective reaction to tissue dysfunction.^27^ Proinflammatory cytokines including TNF‐α and IL‐1β are closely related to the development and progression of mastitis.^34^, ^35^ IL‐1β is produced in the early stages of infection and is thought to be an important mediator of inflammation.^36^ TNF‐α is a pluripotent and proinflammatory cytokine produced by activated macrophages, and TNF‐α induces the production of other cytokines, such as IL‐6, during infection, thereby increasing leukocyte accumulation and amplifying the inflammatory cascade.^37^, ^38^ However, these cytokines can also induce overactivation of neutrophils in tissues, which in turn exacerbates disease process in the host.^39^ The data herein showed that TNF‐α, IL‐1β, neutrophil infiltration and pathological damage were increased in mammary gland. Nevertheless, SSA obviously decreased the level of inflammatory marker including TNF‐α, IL‐1β and MPO activity and alleviated mammary pathological damage. In addition, increased inflammatory cytokines can contribute to barrier damage and TJ destruction.^27^, ^28^, ^29^ Consistently, we found that *S. aureus* decreased the content of TJ protein including ZO‐1, occludin and claudin‐3 in mammary gland, while SSA significantly reversed this change. NF‐κB signalling pathway plays a core role in regulating inflammatory response. The phosphorylation and nuclear translocation of p65 are considered to be a maker of initiation of NF‐κB pathway.^40^ Then, with the degradation of IκB, NF‐κB p65 is released and translocates into the nucleus to bind to target genes and promote cytokine production.^40^ Our results showed that SSA inhibited the phosphorylation of p65 and IκB in *S. aureus*‐induced mammary inflammatory response. These results suggest SSA plays a protective role in *S. aureus*‐induced mastitis by inhibiting inflammatory response and blood‐milk barrier damage.

Cell death is a biological phenomenon, including apoptosis, necrosis and ferroptosis. It is very important to regulate cell death for maintaining normal physiological functions and preventing the onset of diseases.^41^ Ferroptosis, a new form of cell death, is accompanied by mitochondrial morphological changes, including decreased or vanished mitochondria cristae, a ruptured outer mitochondrial membrane and a condensed mitochondrial membrane.^18^, ^42^ The amino acid antiporter system xc^−^ is responsible for GSH, which can influence the expression or activity of glutathione peroxidase 4 (GPX4). GPX4 acts as a phospholipid hydroperoxidase to reduce the production of phospholipid hydrogen peroxide, thereby limiting lipid peroxidation and ferroptosis.^43^, ^44^ Lipid peroxidation plays a key role in the process of ferroptosis, and the end product of lipid peroxidation MDA causes abnormal covalent modifications in proteins and nucleic acids, thereby initiating the cell death programme.^17^, ^45^, ^46^ Iron accumulation is another feature of ferroptosis.^47^, ^48^ Fe^2+^ is an important regulatory factor of oxidative stress and metabolic processes, and excessive accumulation of Fe^2+^ will lead to Fenton reaction, which ultimately causes ferroptosis.^43^ In the present study, we found that SSA significantly upregulated the level of GPX4 and GSH and downregulated PTGS2 and MDA caused by *S. aureus*. Similarly, the content of total Fe^2+^ decreased in the SSA + *S. aureus* group compared with control group. Hence, we speculated that SSA might inhibit ferroptosis, thus alleviating *S. aureus*‐induced mastitis.

SIRT1 is a nicotinamide adenine dinucleotide (NAD)‐dependent histone deacetylase that regulates key metabolic processes including oxidative stress, ageing and apoptosis through the deacetylation of various substrates. Numerous studies have shown that nuclear factor erythroid 2‐related factor 2 (Nrf2) is an important downstream target of SIRT1 signalling.^49^ It is a transcription factor responsible for regulating the redox balance and protective antioxidant activity in mammalian cells. Under pathological conditions, it can transfer to the nucleus, bind to antioxidant response elements (ARE) and drive the expression of its target genes, such as heme oxygenase 1 (HO‐1). HO‐1 and its metabolites can prevent excessive oxidation of lipids and proteins by scavenging hydroxyl radicals, singlet oxygen and superoxide anions, thereby playing an effective role in antioxidant and anti‐apoptosis. Therefore, SIRT1/Nrf2/HO‐1 is an important way to maintain redox balance in vivo. Recent studies demonstrated that SIRT1/Nrf2 signalling was involved in the regulation of ferroptosis. Therefore, the effects of SSA on Nrf2 signalling pathway were measured. We found SSA could activate SIRT1/Nrf2 signalling, and SIRT1 inhibitor could reverse the inhibition of SSA on inflammation and ferroptosis.

In conclusion, our study demonstrates that SSA could alleviate *S. aureus*‐induced mastitis in mice by inhibiting ferroptosis and inflammation via the SIRT1/Nrf2 pathway. These findings suggest that SSA may be a potential therapeutic agent for the treatment of *S. aureus*‐induced mastitis.

## AUTHOR CONTRIBUTIONS

**Lihua Zhao:** Investigation (equal); methodology (equal); project administration (equal); software (equal); supervision (equal); writing – original draft (equal). **Lei Jin:** Investigation (equal); methodology (equal); resources (equal); supervision (equal); validation (equal); visualization (equal). **Bin Yang:** Conceptualization (equal); funding acquisition (equal); investigation (equal); methodology (equal); supervision (equal); validation (equal); writing – review and editing (equal).

## CONFLICT OF INTEREST STATEMENT

The authors have no relevant financial or non‐financial interests to disclose.

## CONSENT FOR PUBLICATION

All authors agree to publish in Journal of Cellular and Molecular Medicine.

## Data Availability

The data that support the findings of this study are available from the corresponding author upon reasonable request.
